# Beyond Trauma Exposure and Symptoms: Preconception Transdiagnostic Vulnerability and Postpartum Outcomes in Healthy Women

**DOI:** 10.3390/jcm15187104

**Published:** 2026-09-13

**Authors:** Keren Avirame, Amit Klein, Sharon Florentin, Moriah Azoulay, Adi Klein, Tamuz Weinberger, Noga Gross, Oren Tene, Atira Bick, Netta Levin, Shiri Ben-David, Inbal Reuveni

**Affiliations:** 1 Psychiatry Division, Tel Aviv Sourasky Medical Center, Tel Aviv 6423906, Israel; amitben95@gmail.com (A.K.); sharonf@tlvmc.gov.il (S.F.); nogagross8@gmail.com (N.G.); orent@tlvmc.gov.il (O.T.); inbalr@tlvmc.gov.il (I.R.); 2 Gray School of Medicine, Tel Aviv University, Tel Aviv 6139001, Israel; 3 Obstetrics and Gynecology Department, Hadassah Hebrew Medical Center, Jerusalem 9112001, Israel; 4 Department of Psychology, The Hebrew University of Jerusalem, Jerusalem 9190501, Israel; moria.azoulay@mail.huji.ac.il (M.A.); adi.klein@mail.huji.ac.il (A.K.); tamuz.weinberger@mail.huji.ac.il (T.W.); shiri.ben-david@mail.huji.ac.il (S.B.-D.); 5 Department of Neurology, Hadassah Hebrew University Medical Center, Jerusalem 9112001, Israel; bica@hadassah.org.il (A.B.); netta@hadassah.org.il (N.L.)

**Keywords:** perinatal mood and anxiety disorders, post-traumatic stress disorder, childhood adversity, emotion dysregulation, learned helplessness, dissociation, transdiagnostic vulnerability, postpartum outcomes, bonding, birth-related trauma symptoms

## Abstract

**Background**: Preconception screening traditionally focuses on physical health indicators and diagnosed psychopathology, potentially overlooking subclinical psychological vulnerabilities that could predict perinatal mental health outcomes. This study examined whether transdiagnostic psychological assessment before pregnancy provides information beyond childhood adversity and current symptom severity. **Methods**: In this prospective cohort study, 170 healthy nulliparous women attempting natural conception underwent preconception assessment and were followed through postpartum. Preconception measures included childhood adversity, transdiagnostic psychological processes (emotional dysregulation, learned helplessness, and dissociation), and psychiatric symptoms (depression, anxiety and post-traumatic stress disorder). Postpartum outcomes included maternal bonding, birth-related trauma symptoms, depression and anxiety levels. Exploratory factor analysis (EFA), structural equation modeling (SEM), and hierarchical linear regressions were used to characterize associations between preconception dimensions and postpartum outcomes. **Results**: Standard trauma screening cutoffs identified 19.3% of women with significant childhood adversity, and over 90% scored below clinical thresholds for current psychopathology. Analyses identified three distinct dimensions: childhood adversity (Risk), transdiagnostic psychological processes (Vulnerability), and current symptoms (Symptoms). Risk was indirectly associated with Symptoms through Vulnerability (β = 0.24), while the direct association between Risk and Symptoms was not significant. Risk and Vulnerability were the primary predictors of maternal bonding (R^2^ = 0.21), whereas Symptoms was the dominant predictor of postpartum anxiety (R^2^ = 0.30), depression (R^2^ = 0.20), and birth-related trauma symptoms (R^2^ = 0.32). **Conclusions**: In a cross-sectional preconception analysis, transdiagnostic psychological vulnerability structurally linked childhood adversity to current symptoms. In a prospective analysis extending from preconception to postpartum, Vulnerability independently predicted postpartum outcomes, including maternal bonding and anxiety. These findings support further investigation of modifiable vulnerabilities, such as emotion dysregulation and learned helplessness, as potential targets for preconception assessment and preventive research.

## 1. Introduction

Perinatal mental health disorders are highly prevalent and have substantial negative effects on both mothers and their children, constituting a major public health issue [1,2]. Mental health before pregnancy, including maternal stress, depression and anxiety, even at subclinical or low levels of depression, have been shown to impact infant neurodevelopment [3,4,5]. Moreover, childhood adversity is consistently linked to perinatal mental health outcomes [6,7], with substantial intergenerational effects [8]. This makes preconception a critical window for optimizing maternal health outcomes [9]. However, preconception and perinatal mental health assessments remain largely symptom- and diagnosis-oriented, while routine preconception care continues to emphasize physical health indicators [10]. Such approaches are important for identifying current distress but may provide limited information about psychological vulnerabilities in women who do not meet clinical thresholds. Whether such assessment provides clinically relevant information beyond conventional symptom- and exposure-based approaches, particularly before pregnancy, remains unclear.

Focusing on trauma exposure may be insufficient, as the relationship between childhood adversity and perinatal psychopathology is multifaceted, with similar trauma histories leading to diverse perinatal outcomes [10]. Even women with significant childhood adversity demonstrate variable perinatal outcomes, with greater resilience associated with reduced postpartum psychopathology and improved maternal wellbeing [11]. Furthermore, it has been shown that the manifestation and relative dominance of specific symptom dimensions fluctuate across perinatal stages, with somatic and sleep symptoms peaking in late pregnancy, while cognitive-affective symptoms may intensify in later postpartum periods [12]. This temporal variability, combined with individual heterogeneity in risk profiles, emphasizes the need for broader assessment throughout the perinatal continuum.

Transdiagnostic psychological processes, viewed as mechanisms that cut across diagnostic categories rather than being specific to a single disorder, have received increasing research attention as potential targets for assessment and intervention [13]. Recent evidence suggests that perinatal symptoms are organized in a dimensional, hierarchical structure, with a general transdiagnostic psychopathology factor accounting for most symptom variation and showing the strongest associations with both adverse and benevolent childhood experiences [14]. Among possible transdiagnostic factors, emotion dysregulation (ED) represents a central mechanism emerging from early adverse experiences [15,16]. ED reflects impairments in the ability to recognize, accept, and effectively manage adverse experiences [17]. ED is associated with postpartum psychiatric symptoms and adverse birth experiences, particularly among individuals with maltreatment histories [18]. Longitudinal and cross-sectional studies demonstrate that ED during pregnancy and postpartum is not only associated with higher levels of depression and anxiety, but also mediates the impact of childhood trauma on perinatal psychopathology [19].

Closely related to ED are beliefs about uncontrollability, conceptually linked to learned helplessness (LH) [20]. The LH theory posits that when individuals experience overwhelming events, they develop expectations that future outcomes will also be uncontrollable, leading to passive coping and increased vulnerability to psychopathology [21]. Such beliefs also predict maladaptive emotion regulation strategies and clinical symptoms [22]. Although LH has received little attention in perinatal populations, it is a well-established process implicated in the onset and maintenance of mood and anxiety disorders and in maladaptive responses following trauma [20]. Notably, maternal LH, manifested as low sense of agency and perceived control, predicts both child behavioral helplessness and depressive symptoms longitudinally, and impairs maternal effective coregulation of children’s emotions [23].

Dissociation represents a third transdiagnostic pathway [24] that often emerges as a dysfunctional emotion regulation strategy in response to overwhelming stress [25], and predicts later psychopathology [26]. Dissociative symptoms, ranging from mild detachment to more severe disruptions in continuity of experience, are also associated with ED [27]. Pregnant women with history of childhood trauma exhibit more dissociative symptoms and peripartum post-traumatic stress disorder (PTSD), with peripartum dissociation and PTSD mediating the association between childhood trauma and both maternal bonding and self-efficacy [28]. Moreover, maternal dissociation prospectively predicts parenting difficulties including impaired bonding, elevated parenting stress, and reduced maternal sensitivity in mother-infant interactions [25].

ED, LH and dissociation represent candidate transdiagnostic dimensions of psychological vulnerability, although their distinct contributions and potential added values beyond conventional preconception screening have not been established. The present study examined whether transdiagnostic preconception assessment of these dimensions could identify vulnerability not captured by trauma history and current symptom severity. Because a shared underlying vulnerability factor can give rise to multiple, divergent outcomes [29], we examined whether these candidate transdiagnostic processes form a coherent joint dimension rather than treating each in isolation. Using exploratory factor analysis, structural equation modeling, and hierarchical multivariate regression in a cohort of healthy women assessed at preconception and followed through postpartum, we investigated: (1) whether adverse childhood experiences, transdiagnostic psychological processes (ED, LH and dissociation), and clinical symptoms (depression, anxiety and PTSD) encompass distinct latent dimensions at preconception; (2) how these latent dimensions relate to one another structurally, and whether one dimension may account for the association between the others; and (3) whether the proximal and distal dimensions of preconception risk show distinct association with postpartum outcomes (maternal bonding, depression, anxiety and birth-related trauma). We hypothesized that transdiagnostic factors, such as ED, LH and dissociation, would emerge as distinct a dimension of psychological vulnerability independent from trauma exposure level and current symptom severity, and would be negatively associated with clinically meaningful postpartum outcomes, including maternal bonding and symptoms of depression, anxiety, and birth-related trauma.

## 2. Methods

### 2.1. Participants

The study recruited healthy nulliparous women aged 18–40 years who were attempting to conceive naturally and willing to participate in a longitudinal study. Exclusion criteria included: (1) lifetime history of psychotic disorder or bipolar disorder; (2) major depressive episode within the past year or current psychiatric treatment; (3) alcohol, tobacco, or substance abuse/dependence within the six months prior to enrollment; (4) medical disorders associated with increased risk of pregnancy complications; (5) requirement for fertility treatments; (6) use of medications other than prenatal vitamins. The Hadassah-Hebrew University Medical Center Ethics Committee approved this study (Protocol No. 0301-20-HMO; approval date: 18 August 2020), and all participants provided written informed consent. Participants received compensation for transportation and time. See Table 1 for complete sample description. 

### 2.2. Procedure

**Figure 1:**
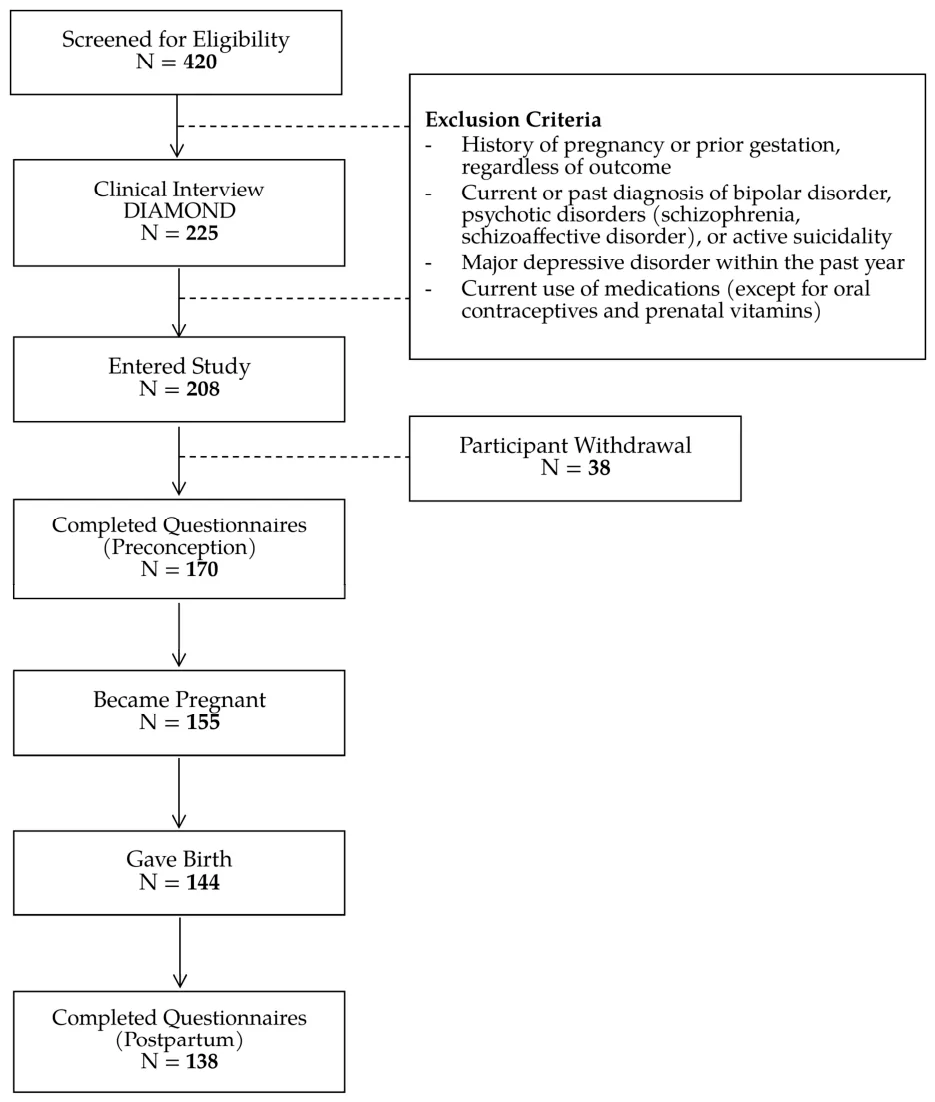
**Study Flow.** Study flow diagram illustrating participant recruitment and retention across study phases. Participants were screened for eligibility and completed a clinical interview using a validated semi-structured diagnostic interview. Those who met inclusion criteria entered the study and completed preconception questionnaires. Participants were subsequently followed through pregnancy and postpartum.

Ethical approval was granted in August 2020 and recruitment began in November 2020. 420 women in the preconception planning stage were recruited through advertisements at community women’s health centers and social media. Women who met criteria and were willing to participate in a longitudinal study were invited to an initial evaluation meeting, where they received an explanation of the study and, if willing to proceed, provided written informed consent. Eligibility was then assessed by a clinician via the Diagnostic Interview for Anxiety, Mood, and Obsessive–Compulsive and Related Neuropsychiatric Disorders (DIAMOND, [30]), administered by a clinician. Between November 2020 and November 2024, 225 completed the DIAMOND interview. The most common reason for exclusion at this stage included current and prior psychiatric diagnoses. Following the interview, 208 women met eligibility criteria and were enrolled in the study; eligible women completed self-report questionnaires administered as digital questionnaires via a HIPAA-compliant secure platform such as REDcap [31]. Out of the 208 women, 170 participants fully completed preconception clinical assessments. The study coordinators subsequently contacted participants approximately every 8 weeks for up to 12 months, or until self-reported pregnancy and birth to coordinate ongoing follow-up assessments through pregnancy and postpartum; 138 women who subsequently gave birth completed postpartum assessments. Participants received compensation for their time and efforts at each study assessment. Mean time from preconception assessment to delivery was 486 days (SD = 228), and from delivery to postpartum assessment was 60 days (SD = 29). See Figure 1 for complete study flow.

### 2.3. Clinical Measures

Participants completed validated self-report questionnaires at preconception and at postpartum. All measures were administered digitally using REDCap [31].

**Preconception Measures:**

The Childhood Trauma Questionnaire (CTQ) is a 28-item retrospective self-report measure on a 5-point Likert scale designed to assess five types of childhood trauma: sexual abuse, physical abuse, physical neglect, emotional abuse, and emotional neglect [32]. According to the CTQ manual [33], established cutoffs for moderate-to-severe exposure were used to derive dichotomous indicators of trauma for each subscale: emotional abuse (≥13), physical abuse (≥10), sexual abuse (≥8), emotional neglect (≥15), and physical neglect (≥10). In our analysis, we additionally derived two composite dimensions by summing the relevant subscale scores: Abuse (emotional, physical, and sexual abuse) and Neglect (emotional and physical neglect).

The Parental Bonding Instrument (PBI) is a 25-item self-administered questionnaire that retrospectively measures participants’ perceptions on perceived parenting by their parents before the age of 16 years [34]. The PBI is composed of two parts, the perceptions of perceived parenting by the mother and the father. Parental attitude is evaluated on a 4-point scale (0 to 3 points) for 12 items relating to care and 13 items relating to overprotection.

The Patient Health Questionnaire (PHQ-9) is a 9-item self-report measure which assesses degree of depression severity by Diagnostic and Statistical Manual of Mental Disorders (DSM)-5 criteria [35]. Each item is scored on a 4-point Likert scale ranging from 0 (not at all) to 3 (nearly every day), evaluating symptoms over the past two weeks, with established cutoffs for minimal (0–4), mild (5–9), moderate (10–14), and severe (15–27) depression, and higher scores indicating greater symptom severity.

The General Anxiety Disorder Questionnaire (GAD-7) is a 7-item self-report measure designed to assess the severity of generalized anxiety symptoms over the past two weeks [36]. Items are rated on a 4-point Likert scale from 0 (not at all) to 3 (nearly every day), with established cutoffs for minimal (0–4), mild (5–9), moderate (10–14), and severe (15–21) anxiety, and higher scores indicating greater symptom severity.

The Post-Traumatic Stress Disorder (PTSD) Checklist for DSM–5 (PCL-5) is a 20-item self-report instrument assessing the severity of PTSD symptoms experienced in the past month according to DSM-5 criteria [37]. Items are rated on a 5-point Likert scale from 0 (not at all) to 4 (extremely), with higher scores reflecting greater PTSD symptom severity. Total scores range from 0 to 80, with a cutoff score of ≥31 suggested for probable PTSD diagnosis.

The Difficulties in Emotion Regulation Scale (DERS) is a 36-item self-report questionnaire designed to assess clinically relevant difficulties in emotion regulation such as engaging in goal-directed behavior, impulse control, and emotional awareness [17]. Responses are rated on a 5-point Likert scale from 1 (almost never) to 5 (almost always), with higher scores indicating greater difficulties in emotion regulation.

The Dissociative Experiences Scale (DES) is a 28-item self-report questionnaire to measure dissociative symptoms [38]. Each item describes a dissociative experience (e.g., amnesia, depersonalization, derealization) and respondents indicate the percentage of time they experience each phenomenon on a scale from 0% (never) to 100% (always), in 10% increments. The total score reflects the overall tendency to dissociate, with higher scores indicating more frequent dissociative experiences.

The Learned Helplessness Questionnaire (LHQ) is a 20-item self-report measure designed to assess various psychological components of learned helplessness, such as perceived control over problems, beliefs about self-efficacy, and subjective well-being and success compared to others [39]. Responses are rated on a 4-point Likert scale ranging from 1 (not at all true) to 4 (very true), with higher scores indicating greater levels of learned helplessness.

**Postpartum Measures**:

The Maternal Postnatal Attachment Scale (MPAS) is a 19-item self-report measure assessing the quality of maternal attachment to the infant during the postpartum period, including quality of attachment, absence of hostility, and pleasure in interaction [40]. Items are scored on two-, four-, or five-point scales ranging from 1 (low attachment) to 5 (high attachment), with higher scores reflecting stronger maternal attachment.

The Birth Trauma Scale (BiTS) is a 29-item self-report questionnaire developed based on DSM-5 criteria to assess postpartum post-traumatic stress disorder (PTSD) symptoms related to childbirth [41]. It includes 22 items measuring PTSD symptom frequency over the past week, rated on a 4-point Likert scale ranging from 0 (not at all) to 3 (five times or more), with higher scores indicating greater symptom severity. Additional items assess subjective birth experience and symptom impact.

Depressive and anxiety symptoms during the postpartum period were reassessed using the PHQ-9 and GAD-7, respectively, both established screening measures used in perinatal populations and included in current American College of Obstetricians and Gynecologists (ACOG) recommendations for perinatal mental health screening [42].

### 2.4. Statistical Analysis

Total scores for clinical measures were calculated according to established norms. Completeness of preconception data ranged from 164/170 to 168/170 across individual study variables and was handled using listwise deletion, yielding a final analytic sample of 160 of 170 participants with complete data. Sample characteristics and clinical measures were summarized using means, standard deviations, and frequency distributions. Clinical symptom severity was categorized according to established cutoffs and was reported descriptively only, to characterize sample symptom severity. All analyses used continuous scores for all measures. Normality was assessed with Shapiro–Wilk tests. Pearson correlations examined bivariate relationships between clinical measures. Internal consistency reliability for each clinical measure was assessed using Cronbach’s α and McDonald’s ω.

Exploratory factor analysis (EFA) was conducted on ten clinical scores (PHQ-9, GAD-7, PCL-5, CTQ Abuse, CTQ Neglect, PBI Maternal Care, PBI Paternal Care, DERS, DES, and LHQ) using maximum likelihood extraction with oblimin rotation, allowing factors to correlate [43]. Prior to conducting the EFA, the suitability of the data for factor analysis was assessed using Bartlett’s test of sphericity and the Kaiser–Meyer–Olkin (KMO) measure of sampling adequacy. The number of factors was determined using parallel analysis, supported by theoretical interpretability and model fit indices (RMSEA—Root Mean Square Error of Approximation ≤ 0.08, Tucker–Lewis Index—TLI ≥ 0.90). Factor loadings, uniqueness values, variance explained by each factor, and inter-factor correlations were examined. Factor scores were extracted and saved for subsequent analyses.

Structural equation modeling (SEM) was used to examine structural associations among latent factors identified in the EFA [44]. The model was estimated using maximum likelihood with Satorra–Bentler mean-adjusted correction to account for non-normality, providing robust standard errors and fit indices [45]. Model fit was evaluated using robust fit indices (CFI—Comparative Fit Index, TLI, RMSEA with 90% confidence intervals, SRMR—Standardized Root Mean Square Residual). Standardized path coefficients (β) and 95% confidence intervals were reported. Indirect effects were tested to examine statistical mediation among latent factors, with follow-up exploratory analyses for measure-level mediation.

Of the original 170 participants, 138 completed the postpartum assessment. Restricting the postpartum analyses to the 160 women included in the preconception analytic sample resulted in sample sizes ranging from 130 to 134 across postpartum measures (130/138 for MPAS and BiTS, 133/138 for PHQ-9, and 134/138 for GAD-7). Hierarchical linear regression examined the three EFA factors as predictors of postpartum outcomes (MPAS, BiTS, GAD7, PHQ-9), with predictors entered sequentially (Risk, then Vulnerability, then Symptoms) to assess incremental variance explained (ΔR^2^) [46]. R^2^ values were interpreted using Cohen’s conventional cutoff [47] for explained variance ranging from small (~0.02), medium (~0.13) to large (~0.26). Multicollinearity and influential cases were evaluated using variance inflation factors (VIF < 5), tolerance values (> 0.20), and Cook’s Distance (D < 1). To assess whether variability in the timing of the postpartum assessment was a confounder, we additionally conducted a sensitivity analysis entering days since birth as a predictor following Risk, Vulnerability, and Symptoms in each regression model. Alpha was set at *p* < 0.05 for all analyses, which were conducted using Jamovi (version 2.7, jamovi project 2026, https://www.jamovi.org) and relevant statistical packages.

## 3. Results

### 3.1. Clinical Characteristics of the Sample

Participants demonstrated low levels of current symptoms, with mean scores well below clinical thresholds across all measures. Over 90% of participants scored in the minimal-to-mild range for depression and anxiety, and only 2.9% exceeded the probable PTSD threshold. Despite the healthy sample characteristics, 19.3% of participants exceeded the CTQ total cutoff for significant childhood trauma. Age and years of education showed no significant correlations with any clinical measures, indicating that observed mean scores were not affected by demographic factors, which were excluded from subsequent analyses. Internal consistency reliability was acceptable-to-good across all measures, as assessed by Cronbach’s alpha and McDonald’s omega (see Table 2). 

### 3.2. Relationships Between Clinical Measures

Correlational analyses were conducted to examine relationships among clinical measures (see Table 3). Measures within similar domains showed strong positive intercorrelations, such as childhood neglect and abuse (r = 0.63, *p* < 0.001), symptoms of depression and anxiety (r = 0.69, *p* < 0.001), and transdiagnostic psychological processes such as emotional regulation and learned helplessness (r = 0.53, *p* < 0.001). Parental care demonstrated strong negative correlations with childhood trauma (r = −0.41 to −0.69, *p* < 0.001).

### 3.3. Exploratory Factor Analysis (EFA)

EFA was conducted on ten clinical measures (PHQ-9, GAD-7, PCL-5, CTQ Abuse, CTQ Neglect, PBI Maternal Care, PBI Paternal Care, DERS, DES, and LHQ) using maximum likelihood extraction with oblimin rotation, allowing factors to correlate. Bartlett’s test of sphericity was significant (χ^2^(45) = 582, *p* < 0.001), and the overall KMO measure of sampling adequacy was 0.75 (item-level range: 0.70–0.86), confirming the data were suitable for factor analysis. The number of factors was determined using parallel analysis, supported by theoretical interpretability and model fit indices. Parallel analysis supported retention of three factors: observed eigenvalues for the first three factors exceeded their corresponding simulated eigenvalues, while the fourth factor’s eigenvalue did not (see Supplementary Figure S1). A three-factor solution demonstrated acceptable fit (RMSEA = 0.05, TLI = 0.96, χ^2^(18) = 25.7, *p* = 0.11) and yielded a clear, interpretable structure explaining 55.5% of total variance. The three-factor solution revealed distinct but related constructs (see Table 4). Factor 1 (23.8% of variance) represented early adverse experiences and lack of parental care and was labeled “Risk”. Factor 2 (17.4% of variance) captured present-day clinical distress and was labeled “Symptoms”. Factor 3 (14.4% of variance) encompassed underlying psychological processes affecting coping and regulation capacities, labeled “Vulnerability”. Inter-factor correlations were small to moderate, with the strongest association observed between Symptoms and Vulnerability (r = 0.42), followed by correlations between Risk and Vulnerability (r = 0.30) and Risk and Symptoms (r = 0.19).

In summary, the identified three dimensions will be referred throughout the subsequent analyses as “Risk”, representing developmental-contextual adversity (childhood abuse/neglect experiences and caregiving context); “Vulnerability”, representing transdiagnostic psychological processes related to ED, LH, and dissociation; and “Symptoms”, representing current depression, anxiety, and PTSD symptom burden. It is important to note that ‘Risk,’ ‘Vulnerability,’ and ‘Symptoms’ denote the three EFA-derived dimensions descriptively and are not intended as formal risk-factor classifications [48].

### 3.4. Structural Equation Model (SEM)

**Figure 2:**
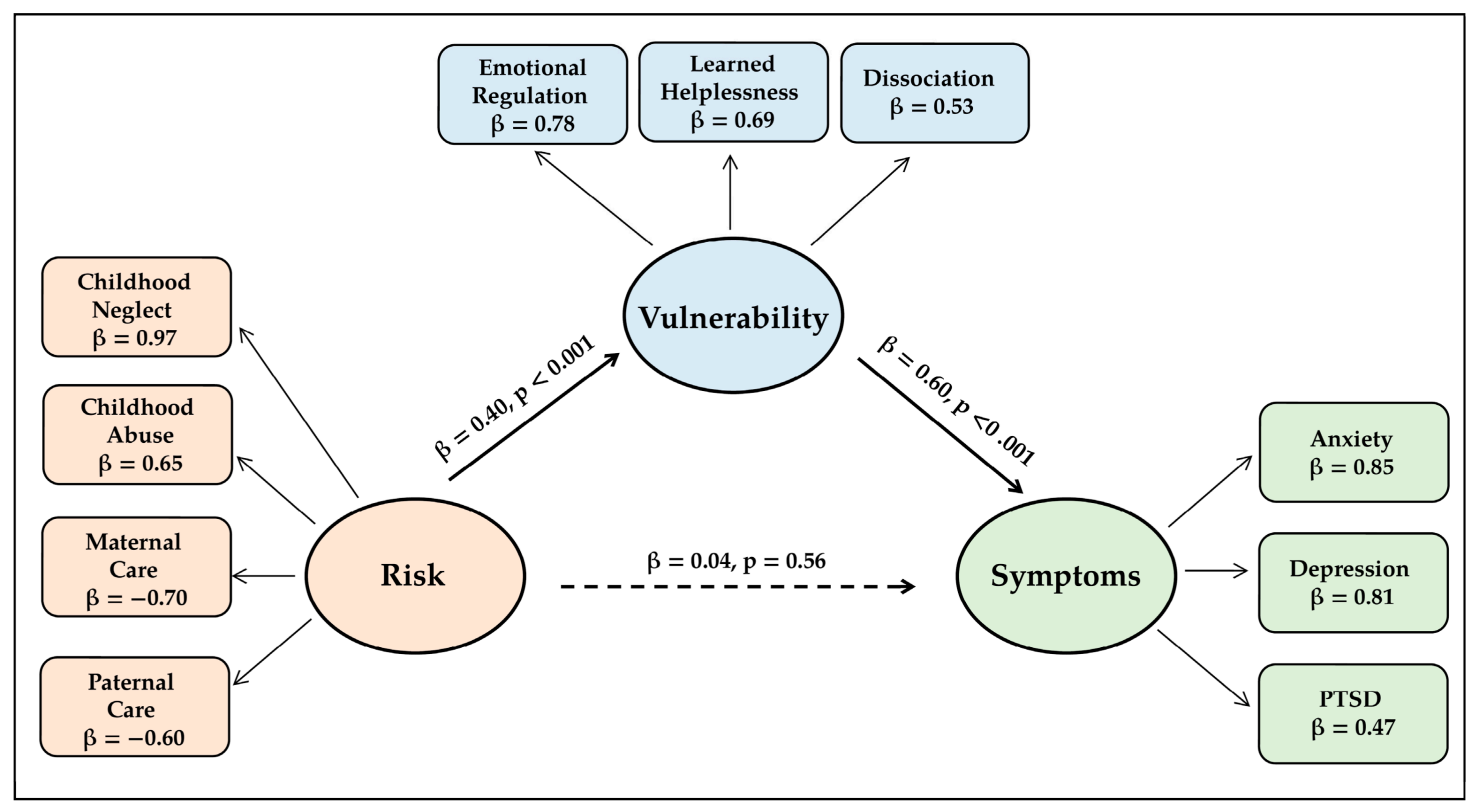
**Structural Associations between Risk, Vulnerability, and Symptoms at Preconception.** Standardized path coefficients from the structural equation model examining relationships among latent Risk (Orange), Vulnerability (Blue), and Symptoms (Green) factors. Solid lines indicate significant paths; dashed lines indicate nonsignificant paths. All factor loadings were significant (*p* < 0.001). The indirect effect of Risk on Symptoms through Vulnerability was significant (β = 0.24, *p* = 0.001). Values within boxes represent standardized factor loadings. CTQ, Childhood Trauma Questionnaire; PBI, Parental Bonding Instrument; DERS, Difficulties in Emotion Regulation Scale; LHQ, Learned Helplessness Questionnaire; DES, Dissociative Experiences Scale; GAD-7, Generalized Anxiety Disorder; PHQ-9, Patient Health Questionnaire; PCL-5, PTSD Checklist for DSM-5.

Using SEM, we examined the relationships between the three latent constructs identified in the EFA: Risk, Vulnerability, and Symptoms. The model was estimated using robust maximum likelihood (MLM) with Satorra–Bentler mean-adjusted correction to account for non-normality. The model demonstrated acceptable fit to the data based on robust fit indices, χ^2^_SB(32) = 51.3, *p* = 0.02; CFI = 0.952; TLI = 0.93; RMSEA = 0.07 (90% CI [0.03, 0.11]), *p* = 0.16; SRMR = 0.07. All observed indicators loaded significantly onto their respective latent variables, with standardized factor loadings ranging from 0.47 to 0.97, supporting adequate measurement of the latent constructs (see Figure 2 for model visualization and Supplementary Table S1 for full measurement model parameters).

At the structural level, Risk was significantly associated with Vulnerability (β = 0.40, 95% CI [0.24, 0.56], *p* < 0.001), and Vulnerability was strongly associated with Symptoms (β = 0.60, 95% CI [0.41, 0.79], *p* < 0.001). The direct path from Risk to Symptoms was not significant (β = −0.05, 95% CI [−0.21, 0.12], *p* = 0.57). A significant indirect effect of Risk on Symptoms through Vulnerability was observed (β = 0.24, 95% CI [0.11, 0.37], *p* = 0.001), consistent with statistical mediation. Follow-up exploratory analyses examined each Vulnerability indicator individually as a mediator of the Risk–Symptoms association. LH demonstrated the largest indirect effect (indirect effect: β = 0.16, 95% CI [0.07, 0.25], *p* = 0.002), followed by ED (indirect effect: β = 0.10, 95% CI [0.03, 0.18], *p* = 0.01), and dissociation (indirect effect: β = 0.05, 95% CI [0.00, 0.09], *p* = 0.06). Notably, the combined indirect effect of Vulnerability (β = 0.24) exceeded that observed for any individual component, suggesting that the joint Vulnerability construct may better capture the observed pattern of associations.

### 3.5. Associations and Reliability of Postpartum Clinical Measures

Internal consistency reliability was good across all postpartum measures. Maternal bonding showed good reliability (α = 0.83, ω = 0.86), as did birth-related trauma symptoms (α = 0.85, ω = 0.85), postpartum anxiety symptoms (α = 0.87, ω = 0.88), and postpartum depressive symptoms (α = 0.80, ω = 0.82). Correlational analyses were conducted to examine relationships among postpartum clinical outcomes (see Table 5 for full results). Measures reflecting current symptoms showed strong positive intercorrelations, including depression with anxiety (r = 0.73, *p* < 0.001) and depression with birth-related trauma symptoms (r = 0.71, *p* < 0.001), as well as anxiety with birth-related trauma symptoms (r = 0.66, *p* < 0.001). In contrast, maternal–infant bonding demonstrated moderate negative correlations with current symptoms and birth-related trauma (r = −0.48 to −0.53, *p* < 0.001).

### 3.6. Predicting Postpartum Outcomes

To examine the predictive validity of the latent factors, hierarchical linear regressions were conducted predicting maternal bonding (MPAS), birth-related trauma symptoms (BiTS), postpartum depression (PHQ-9), and postpartum anxiety (GAD-7) (see Table 6 for results). Predictors were entered sequentially in all models (Risk, Vulnerability, Symptoms). For maternal bonding, the final model was significant (R^2^ = 0.21, medium-to-large effect), with both Risk and Vulnerability emerging as significant predictors, whereas Symptoms did not contribute additional explained variance. In contrast, for birth trauma symptoms, only the Symptoms factor remained significant in the final model (R^2^ = 0.32, large effect), with earlier Risk and Vulnerability effects attenuated after the Symptoms factor was included. A similar pattern was observed for postpartum depression (R^2^ = 0.20, medium-to-large effect) and anxiety (R^2^ = 0.30, large effect), in which the Symptoms factor accounted for the largest proportion of explained variance, while Risk was no longer significant. As for Vulnerability, it showed smaller or residual effects for postpartum depression, and independent predictive value for postpartum anxiety even after accounting for current symptom burden. Across outcomes, Risk and Vulnerability were more strongly associated with maternal bonding, whereas postpartum psychopathology outcomes were related to current symptoms burden. Full regression statistics for each step, including unstandardized coefficients (B, SE), t-values, adjusted R^2^, and F-change statistics, are presented in Supplementary Table S2.

To examine whether variability in the timing of the postpartum assessment (days since birth) confounded these associations, we conducted a sensitivity analysis with days since birth as an additional predictor following Risk, Vulnerability, and Symptoms. Days since birth was not a significant predictor of any postpartum outcome and did not meaningfully improve model fit (maternal bonding: ΔR^2^ = 0.001, F(1125) = 0.15, *p* = 0.70; birth-related trauma symptoms: ΔR^2^ = 0.003, F(1125) = 0.57, *p* = 0.45; depression: ΔR^2^ < 0.001, F(1127) = 0.02, *p* = 0.88; anxiety: ΔR^2^ < 0.001, F(1128) = 0.05, *p* = 0.82), and the pattern of associations remained unchanged.

## 4. Discussion

Using a data-driven analytic framework in a preconception sample of healthy women, this study identified three distinct dimensions of mental health risk: childhood experiences (Risk), transdiagnostic psychological processes (Vulnerability), and current distress (Symptoms). SEM at preconception identified a significant indirect association between Risk and Symptoms through Vulnerability, while the direct association between Risk and Symptoms was not significant. Notably, this pattern emerged in a cohort in which over 90% of participants scored below clinical thresholds and only 19.3% exceeded standard trauma cutoffs, a population that current screening practices would largely overlook. Our findings extend prior evidence that childhood adverse experiences exert their effects on adult psychopathology through psychological processes such as ED [49,50] and intolerance of uncertainty [51], by showing that these associations between childhood adversity, transdiagnostic vulnerability, and current symptoms are already detectable at the preconception stage.

The three Vulnerability dimensions contributed to the observed indirect association, although with notable differences in effect size and statistical significance. LH emerged as the strongest individual mediator, followed by ED, while dissociation fell short of statistical significance. Importantly, the combined indirect effect exceeded that of any individual mediator, suggesting that considering these vulnerability dimensions may better jointly capture the indirect pathway from childhood adversity to current symptoms. This pattern is consistent with both theoretical and empirical associations between the three constructs: ED and LH both related to experiences of overwhelming and uncontrollable stress, yet capture distinct facets of vulnerability, e.g., the capacity to manage emotional states versus the expectation that one can influence outcomes. On the other hand, dissociation frequently co-occurs with, and is partly explained by, ED [27], which may account for its weaker contribution when modeled alongside the other two dimensions.

The prominence of LH is particularly noteworthy given its understudied role in perinatal populations. An individual’s sense of control may be especially challenged during the transition to parenthood, a period inherently characterized by uncertainty, shifting roles, and new responsibilities. In fact, higher levels of control have been associated with lower psychological distress among new parents [52]. Conversely, perceived loss of control during childbirth predicted poorer postpartum well-being, especially among women who were high in intolerance of uncertainty [53]. LH may also be relevant to intergenerational outcomes; maternal uncontrollability has been indirectly linked in previous research to both maternal depression and child helplessness over time [23].

Another key finding of this study was the differential prediction of postpartum outcomes. Risk and Vulnerability were the primary predictors of maternal bonding, whereas Symptoms predicted postpartum depression, anxiety, and birth-related trauma symptoms. This corresponds with previous research demonstrating that early mother-child interactions and bonding are shaped by early experiences, caregiving history and transdiagnostic psychological processes [54]. However, postpartum psychopathology showed stronger association with preconception Symptoms, consistent with the well-established continuity of psychiatric symptoms across the perinatal period [12]. A recent meta-analysis of 99 samples (N = 110,968) found that maternal depression, anxiety, and overall stress strongly correlate with poorer maternal bonding [55], as also observed in our postpartum associations. However, in our cohort when Risk, Vulnerability, and Symptoms were modeled hierarchically, current Symptoms no longer independently predicted bonding.

Notably, Vulnerability retained independent predictive value for postpartum anxiety even after accounting for Symptoms, suggesting that transdiagnostic processes carry incremental predictive value beyond symptom screening for this outcome. This finding is particularly relevant given that research on postpartum anxiety among women with childhood adversity remains limited. A recent study demonstrated that adversity predicts elevated anxiety across a three-year postpartum period based on a categorical adversity threshold (≥4 childhood adverse experiences) [56]. Our findings extend this by showing that transdiagnostic Vulnerability independently predicted postpartum anxiety, not Risk, and that this association can be detectable even among women who fall below standard trauma cutoffs. Our finding is consistent with evidence linking maladaptive cognitive-emotional processes to increase susceptibility to psychopathology [57]. Moreover, it supports further investigation of whether identifying and targeting these processes at preconception could provide a useful window for intervention.

The current findings may have implications for future approaches to preconception assessment and intervention, although their translation into clinical practice requires further investigation. First, the differential prediction of postpartum outcomes may have implications for current perinatal screening approaches. Our findings suggest that symptom-based assessment alone may not capture all factors associated with later maternal bonding difficulties. This accords with emerging evidence that subgroups of individuals with low symptom severity can show elevated psychological risk that traditional measures fail to detect [58]. Future investigation of whether incorporating transdiagnostic vulnerability measures into preconception assessment could improve early identification of women at risk for later bonding difficulties, particularly given that maternal bonding predicts children’s socio-affective outcomes [59], is warranted. Second, the Vulnerability dimensions identified here may represent candidate modifiable targets for future preventive research. ED can be addressed through interventions that strengthen emotion regulation competencies [60], while approaches targeting perceived control and agency may be relevant to LH [61]. Dissociative tendencies may similarly be addressed through grounding, stabilization, and trauma-focused approaches when clinically indicated [62]. Given that the present study did not test whether modifying these processes could change perinatal outcomes, future intervention studies are needed to determine whether targeting these vulnerabilities before pregnancy can reduce subsequent risk.

Several limitations should be considered when interpreting these findings. First, the sample consisted of young, healthy, predominantly Jewish women with relatively high educational attainment, and excluded women with recent major depression, current psychiatric treatment, or substance use. This selection likely constrained variability in both symptom burden and risk exposure and may have influenced the magnitude of the observed associations. It also limits generalizability to more socioeconomically and culturally diverse or under-resourced populations, and to women with psychiatric or medical comorbidities, while restricting our ability to examine interactions between physical and psychological preconception risk. Future studies should include more heterogeneous populations across socioeconomic, cultural, psychiatric, and medical characteristics. Second, all measures relied on self-report, introducing the possibility of recall bias and social desirability effects, particularly for retrospective assessments of childhood adversity. Third, while the sample size was adequate for the primary analyses, it is modest relative to large epidemiological cohort studies, particularly given the breadth of variables assessed. Fourth, the three-factor structure and structural model were derived and tested in the same sample, without cross-validation in an independent cohort; replication in larger, independent samples is needed to confirm the stability of this factor architecture. Fifth, the SEM relating Risk, Vulnerability, and Symptoms was based on constructs assessed at preconception, so the indirect effects reflect statistical associations rather than temporal or causal mediation. Longitudinal and intervention studies are needed to test whether vulnerability precedes symptoms and whether modifying it before pregnancy affects postpartum outcomes. Finally, the selection of transdiagnostic measures was guided by our theoretical framework, and other relevant constructs, such self-efficacy or mentalization, were not assessed. Nonetheless, the measures included reflect established targets of evidence-based interventions [63] underscoring the clinical relevance of these findings.

## 5. Conclusions

This study highlights the potential value of extending preconception mental health assessment beyond childhood adversity and current symptoms to include transdiagnostic psychological vulnerability. Vulnerability statistically accounted for the indirect association between childhood adversity and current symptoms in a cross-sectional analysis at preconception and predicted postpartum outcomes, including maternal bonding and anxiety. The current findings suggest that transdiagnostic vulnerability may provide clinically relevant information not captured by symptom assessment alone. Future studies should determine whether incorporating such measures into preconception assessment improves risk identification and whether interventions targeting these processes can improve maternal and infant outcomes.

## Figures and Tables

**Table 1:** Sample Description.

	Preconception (N = 170)		Postpartum (N = 138)	
**Age [years]**	25.22	3.80	25.13	3.81
**Country of birth**				
Israel	150	89.8%	121	89.6%
Other	17	10.2%	14	10.4%
**Education level**				
Secondary education	28	16.9%	25	18.5%
Higher education	138	83.1%	110	81.5%
**Marital status**				
Married	153	91.6%	126	93.3%
Common-law partners	14	8.4%	9	6.7%
**Religious affiliation**				
Jewish	170	100%	138	100%
**Religiosity level**				
Ultra-orthodox	14	8.4%	11	8.2%
Modern orthodox	115	68.9%	95	70.4%
Traditional	13	7.8%	11	8.2%
Secular	23	13.8%	17	12.6%
Other	2	1.2%	1	0.7%
**Household income**				
Much lower than average	62	37.1%	55	40.7%
Slightly lower than average	40	24.0%	34	25.2%
Average	22	13.2%	17	12.6%
Slightly higher than average	32	19.2%	21	15.6%
Much higher than average	11	6.6%	8	5.9%
**Conception method**				
Natural			97	78.9%
Medication-assisted			11	8.9%
Artificial insemination			6	4.9%
In vitro fertilization			9	7.3%
**Childbirth [weeks]**			39.20	1.94
**Parturition type**				
Normal vaginal delivery			100	72.5%
Vacuum extraction			21	15.2%
Scheduled c-section			3	2.2%
Unplanned c-section			14	10.1%
**Baby’s biological sex**				
Male			76	55.1%
Female			62	44.9%
**Baby’s birth weight [grams]**			3202.68	536.72

Demographic, socioeconomic, and obstetric characteristics of participants assessed at preconception (N = 170) and postpartum (N = 138). Continuous variables (age, childbirth and baby’s birth weight) are presented as mean and SD; categorical variables are presented as N and percentage. Postpartum variables include conception method, delivery characteristics, and neonatal outcomes.

**Table 2:** Clinical Characteristics of the Sample at Preconception.

Measure	N	Mean	SD	Range	α	ω	Severity Categories	N	%
**Emotional Regulation (DERS)**	167	71.6	17.7	43–120	0.92	0.92			
**Learned Helplessness (LHQ)**	165	37.1	8.3	22–66	0.90	0.91			
**Dissociation (DES)**	168	6.7	5.3	0–24	0.84	0.86	(≥30)	0	0%
**Depression (PHQ-9)**	167	4.1	3.1	0–17	0.70	0.72	Minimal (0–4)	105	62.9%
							Mild (5–9)	52	31.1%
							Moderate (10–14)	7	4.2%
							Severe (15–27)	3	1.8%
**Anxiety (GAD-7)**	167	2.9	2.7	0–10	0.80	0.80	Minimal (0–4)	123	73.7%
							Mild (5–9)	41	24.6%
							Moderate (10–14)	3	1.8%
							Severe (15–27)	0	0%
**Post-Traumatic Stress** **(PCL-5)**	164	8.8	10.4	0–45	0.92	0.92	Below cutoff (<31)	156	95.1%
							Above cutoff (≥31)	8	4.9%
**Parental Bonding-Maternal Care (PBI)**	167	30.0	6.6	5–36	0.93	0.93			
**Parental Bonding-Paternal Care (PBI)**	167	27.6	8.2	2–36	0.94	0.94			
**Childhood Trauma (CTQ)**	166	32.4	8.8	25–75	0.90	0.90	Below cutoff	134	80.7%
							Above cutoff	32	19.3%
**Childhood Trauma** **(CTQ Subscales)**							Cutoff		
**Emotional Neglect**		8.0	3.5	5–18			(≥15)	16	9.6%
**Emotional Abuse**		7.4	2.8	5–22			(≥13)	9	5.4%
**Physical Abuse**		5.4	1.5	5–20			(≥10)	5	3.0%
**Sexual Abuse**		5.9	2.6	5–25			(≥8)	17	10.2%
**Physical Neglect**		5.8	1.5	5–15			(≥10)	4	2.4%

Distribution of psychiatric symptom severity and childhood trauma exposure at preconception assessment. Values represent mean and SD, observed range, and frequency distribution across established clinical severity categories. Severity classifications are based on validated instrument cutoffs. α and ω represent Cronbach’s alpha and McDonald’s omega, respectively, reflecting internal consistency reliability of each measure in the present sample. DERS, Difficulties in Emotion Regulation Scale; DES, Dissociative Experiences Scale; LHQ, Learned Helplessness Questionnaire; PHQ-9, Patient Health Questionnaire; GAD-7, Generalized Anxiety Disorder; PCL-5, PTSD Checklist for DSM-5; PBI, Parental Bonding Instrument; CTQ, Childhood Trauma Questionnaire.

**Table 3:** Correlations between Clinical Measures at Preconception.

Variable	1	2	3	4	5	6	7	8	9
**1. CTQ Neglect (Childhood Trauma)**									
**2. CTQ Abuse (Childhood Trauma)**	0.63 ***								
**3. PCL-5 (PTSD)**	0.33 ***	0.34 ***							
**4. PHQ-9 (Depression)**	0.12	0.06	0.36 ***						
**5. GAD-7 (Anxiety)**	0.09	0.00	0.39 ***	0.70 ***					
**6. PBI—Bonding (Maternal Care)**	−0.69 ***	−0.41 ***	−0.22 **	−0.24 **	−0.13				
**7. PBI—Bonding (Paternal Care)**	−0.59 ***	−0.44 ***	−0.20 *	−0.08	−0.04	0.40 ***			
**8. DERS (Emotional Regulation)**	0.23 **	0.11	0.18 *	0.34 ***	0.36 ***	−0.23 **	−0.30 ***		
**9. DES (Dissociation)**	0.14	0.17 *	0.17 *	0.24 **	0.27 ***	−0.12	−0.23 **	0.48 ***	
**10. LHQ (Learned Helplessness)**	0.35 ***	0.25 **	0.25 **	0.36 ***	0.37 ***	−0.25 **	−0.33 ***	0.53 ***	0.27 ***

N = 160. CTQ, Childhood Trauma Questionnaire; PBI, Parental Bonding Instrument; GAD-7, Generalized Anxiety Disorder; PHQ-9, Patient Health Questionnaire; PCL-5, PTSD Checklist for DSM-5; DERS, Difficulties in Emotion Regulation Scale; DES, Dissociative Experiences Scale; LHQ, Learned Helplessness Questionnaire. * *p* < 0.05, ** *p* < 0.01, *** *p* < 0.001.

**Table 4:** Three Distinct Dimensions of Mental Health at Preconception.

Variable	Factor 1 (Risk)	Factor 2 (Symptoms)	Factor 3 (Vulnerability)	Uniqueness (ψ)
**CTQ Neglect (Childhood Trauma)**	0.99	−0.02	−0.01	0.04
**CTQ Abuse (Childhood Trauma)**	0.66	−0.10	−0.00	0.58
**PBI Maternal (Parental Care)**	−0.68	−0.02	−0.04	0.50
**PBI Paternal (Parental Care)**	−0.56	0.09	−0.21	0.60
**GAD-7 (Anxiety)**	−0.04	0.85	0.02	0.27
**PHQ-9 (Depression)**	−0.00	0.81	0.01	0.33
**PCL-5 (PTSD)**	0.29	0.45	−0.08	0.70
**DERS (Emotional Regulation)**	−0.00	−0.00	0.96	0.08
**DES (Dissociation)**	0.00	0.10	0.46	0.73
**LHQ (Learned Helplessness)**	0.21	0.22	0.40	0.60

Factor loadings and uniqueness estimates derived from exploratory factor analysis of clinical and psychological measures (N = 160). Factors were interpreted as Risk, Symptoms, and Vulnerability dimensions based on loading structure. Uniqueness (ψ) reflects variance not explained by the common factors. CTQ, Childhood Trauma Questionnaire; PBI, Parental Bonding Instrument; GAD-7, Generalized Anxiety Disorder; PHQ-9, Patient Health Questionnaire; PCL-5, PTSD Checklist for DSM-5; DERS, Difficulties in Emotion Regulation Scale; DES, Dissociative Experiences Scale; LHQ, Learned Helplessness Questionnaire.

**Table 5:** Correlations Among Clinical Measures at Postpartum.

Variable	1	2	3
**1. MPAS (Maternal Bonding)**			
**2. BiTS (Birth-Related Trauma Symptoms)**	−0.48 ***		
**3. PHQ-9 (Depression)**	−0.52 ***	0.71 ***	
**4. GAD-7 (Anxiety)**	−0.53 ***	0.66 ***	0.73 ***

MPAS = Maternal Postnatal Attachment Scale; BiTS = Birth Trauma Scale; PHQ-9 = Patient Health Questionnaire-9; GAD-7 = Generalized Anxiety Disorder-7. *** *p* < 0.001.

**Table 6:** Predicting Postpartum Outcomes by Factors.

Outcome	Model Step	Risk Factor	Vulnerability Factor		Symptoms Factor		Model
		β	β	ΔR^2^	β	ΔR^2^	R^2^
MPAS Maternal Bonding	Step 1	−0.37 ***	-				0.14 ***
	Step 2	−0.28 **	−0.27 **	0.06			0.20 ***
	Step 3	−0.27 **	−0.21 *		−0.13	0.012	0.21 ***
BiTS Traumatic Birth	Step 1	0.21 *	-		-		0.04 *
	Step 2	0.10	0.34 ***	0.10	-		0.14 ***
	Step 3	0.05	0.12		0.48 ***	0.172	0.32 ***
PHQ-9 Postpartum Depression	Step 1	0.19 *	-		-		0.03 *
	Step 2	0.09	0.29 **	0.07	-		0.11 ***
	Step 3	0.07	0.12		0.36 ***	0.094	0.20 ***
GAD-7 Postpartum Anxiety	Step 1	0.21 *	-		-		0.04 *
	Step 2	0.07	0.39 ***	0.13	-		0.18 ***
	Step 3	0.05	0.19 *		0.41 ***	0.125	0.30 ***

Hierarchical linear regression models examining associations between latent Risk, Vulnerability, and Symptoms factors and postpartum outcomes (maternal bonding, traumatic birth symptoms, postpartum depression, and postpartum anxiety). Predictors were entered sequentially: Step 1 = Risk factor; Step 2 = Vulnerability factor; Step 3 = Symptom factor. Values represent standardized regression coefficients (β) from each model step. ΔR^2^ indicates the change in explained variance (ΔR^2^) associated with the entry of each predictor block. R^2^ values represent total variance explained by the model at each step. MPAS, Maternal Postnatal Attachment Scale; BiTS, Birth Trauma Scale; PHQ-9, Patient Health Questionnaire; GAD-7, Generalized Anxiety Disorder. * *p* < 0.05, ** *p* < 0.01, *** *p* < 0.001.

## Data Availability

The data of this study are available from the corresponding author upon reasonable request.

## Supplementary Materials

The following supporting information can be downloaded at: https://www.mdpi.com/article/10.3390/jcm15187104/s1, Figure S1. Scree Plot of Parallel Analysis; Table S1. Structural Equation Model: Standardized Path Coefficients; Table S2. Full Hierarchical Regression Statistics Predicting Postpartum Outcomes.

## References

[1] Rogers A., Obst S., Teague S.J., Rossen L., Spry E.A., Macdonald J.A., Sunderland M., Olsson C.A., Youssef G., Hutchinson D. (2020). Association Between Maternal Perinatal Depression and Anxiety and Child and Adolescent Development: A Meta-Analysis. JAMA Pediatr..

[2] Wisner K.L., Murphy C., Thomas M.M. (2024). Prioritizing Maternal Mental Health in Addressing Morbidity and Mortality. JAMA Psychiatry.

[3] Donnici C., Long X., Reynolds J., Giesbrecht G.F., Dewey D., Letourneau N., Huo Y., Landman B., Lebel C. (2023). Prenatal Depressive Symptoms and Childhood Development of Brain Limbic and Default Mode Network Structure. Hum. Brain Mapp..

[4] Rogers A.M., Youssef G.J., Teague S., Sunderland M., Le Bas G., Macdonald J.A., Mattick R.P., Allsop S., Elliott E.J., Olsson C.A. (2023). Association of Maternal and Paternal Perinatal Depression and Anxiety with Infant Development: A Longitudinal Study. J. Affect. Disord..

[5] Lebel C., Walton M., Letourneau N., Giesbrecht G.F., Kaplan B.J., Dewey D. (2016). Prepartum and Postpartum Maternal Depressive Symptoms Are Related to Children’s Brain Structure in Preschool. Biol. Psychiatry.

[6] Souch A.J., Jones I.R., Shelton K.H., Waters C.S. (2025). Adverse Childhood Experiences (ACEs) and Childbearing and Perinatal Mental Health Outcomes in a Clinical Sample of Women. J. Affect. Disord..

[7] Edwards H., Phillips C., Esterman A., Buisman-Pijlman F., Gordon A. (2017). Risk Factors and Assessment Tools for Mother-Infant Bonding: A Scoping Review to Assist Future Research. Evid. Based Midwifery.

[8] Yehuda R., Lehrner A. (2018). Intergenerational Transmission of Trauma Effects: Putative Role of Epigenetic Mechanisms. World Psychiatry.

[9] Kee M.Z.L., Ponmudi S., Phua D.Y., Rifkin-Graboi A., Chong Y.S., Tan K.H., Chan J.K.Y., Broekman B.F.P., Chen H., Meaney M.J. (2021). Preconception Origins of Perinatal Maternal Mental Health. Arch. Womens Ment. Health.

[10] Sellars E., Oliver B.R., Leijten P., Bowes L. (2025). Trajectories of Psychosocial Functioning across Maltreatment Levels: A Group-Based Modeling Approach to Resilience. Dev. Psychopathol..

[11] Sexton M.B., Hamilton L., McGinnis E.W., Rosenblum K.L., Muzik M. (2015). The Roles of Resilience and Childhood Trauma History: Main and Moderating Effects on Postpartum Maternal Mental Health and Functioning. J. Affect. Disord..

[12] Cochran A.L., Pingeton B.C., Goodman S.H., Laurent H., Rathouz P.J., Newport D.J., Stowe Z.N. (2020). A Transdiagnostic Approach to Conceptualizing Depression across the Perinatal Period in a High-Risk Sample. J. Abnorm. Psychol..

[13] Harvey A., Watkins E., Mansell W., Shafran R. (2004). Cognitive Behavioural Processes Across Psychological Disorders: A Transdiagnostic Approach to Research and Treatment.

[14] Clark H.M., Hankin B.L., Narayan A.J., Davis E.P. (2024). Risk and Resilience Factors for Psychopathology during Pregnancy: An Application of the Hierarchical Taxonomy of Psychopathology (HiTOP). Dev. Psychopathol..

[15] Shipman K., Zeman J., Penza S., Champion K. (2000). Emotion Management Skills in Sexually Maltreated and Nonmaltreated Girls: Adevelopmental Psychopathology Perspective. Dev. Psychopathol..

[16] Weissman D.G., Bitran D., Miller A.B., Schaefer J.D., Sheridan M.A., McLaughlin K.A. (2019). Difficulties with Emotion Regulation as a Transdiagnostic Mechanism Linking Child Maltreatment with the Emergence of Psychopathology. Dev. Psychopathol..

[17] Gratz K.L., Roemer L. (2004). Multidimensional Assessment of Emotion Regulation and Dysregulation: Development, Factor Structure, and Initial Validation of the Difficulties in Emotion Regulation Scale. J. Psychopathol. Behav. Assess..

[18] Wright K.R., Zhou A.M., Molina N.C., De Vos N., Kaliush P.R., Conradt E., Crowell S.E. (2026). A Multimethod Longitudinal Examination of the Effects of Childhood Maltreatment on Birth Experiences and Postpartum Mental Health. Dev. Psychopathol..

[19] Giles L., Khoury J.E. (2025). Emotion Regulation during Pregnancy: A Pathway from Maternal Childhood Maltreatment to Perinatal Mental Health. J. Affect. Disord..

[20] Tafet G.E., Ortiz Alonso T. (2025). Learned Helplessness and Learned Controllability: From Neurobiology to Cognitive, Emotional and Behavioral Neurosciences. Front. Psychiatry.

[21] Abramson L.Y., Seligman M.E., Teasdale J.D. (1978). Learned Helplessness in Humans: Critique and Reformulation. J. Abnorm. Psychol..

[22] Salguero J.M., Ramos-Cejudo J. (2023). A Multi-Study Examination of the Relevance of the Metacognitive Beliefs about Uncontrollability in Emotion Regulation and Clinical Symptoms. J. Affect. Disord..

[23] River L.M., Borelli J.L., Vazquez L.C., Smiley P.A. (2018). Learning Helplessness in the Family: Maternal Agency and the Intergenerational Transmission of Depressive Symptoms. J. Fam. Psychol..

[24] Lyssenko L., Schmahl C., Bockhacker L., Vonderlin R., Bohus M., Kleindienst N. (2018). Dissociation in Psychiatric Disorders: A Meta-Analysis of Studies Using the Dissociative Experiences Scale. AJP.

[25] Williams K., Moehler E., Kaess M., Resch F., Fuchs A. (2022). Dissociation Links Maternal History Of Childhood Abuse To Impaired Parenting. J. Trauma Dissociation.

[26] Liu J., Tan C.M., Ho J.C., Tang C., Verma S., Subramaniam M. (2025). Dissociation as Transdiagnostic Mediator between Trauma Exposure and the Post-Traumatic Stress Disorder-Psychosis Symptom Spectrum: A Structural Equation Modelling Meta-Analysis. J. Affect. Disord..

[27] Bruno S., Tacchino C., Anconetani G., Velotti P. (2025). Unravelling the Associations between Dissociation and Emotion (Dys)Regulation: A Multidimensional Meta-Analytic Review. J. Affect. Disord..

[28] Brenner I., Ginzburg K., Golan A., Igawa M.S., Lurie I., Reicher Y., Talmon A., Tomashev R., Padoa A. (2024). Peripartum Dissociation, Sense of Control, Postpartum Posttraumatic Stress Disorder and Emotional Adjustment to Motherhood in Adult Survivors of Childhood Maltreatment. Arch. Womens Ment. Health.

[29] Nolen-Hoeksema S., Watkins E.R. (2011). A Heuristic for Developing Transdiagnostic Models of Psychopathology: Explaining Multifinality and Divergent Trajectories. Perspect. Psychol. Sci..

[30] Tolin D.F., Bowe W., Davis E., Hannan S., Springer K., Worden B., Steinman S.A. (2016). Diagnostic Interview for Anxiety, Mood, and OCD and Related Neuropsychiatric Disorders (DIAMOND).

[31] Harris P.A., Taylor R., Minor B.L., Elliott V., Fernandez M., O’Neal c L., McLeod L., Delacqua G., Delacqua F., Kirby J. (2019). The REDCap consortium: Building an international community of software platform partners. J. Biomed. Inform..

[32] Bernstein D.P., Stein J.A., Newcomb M.D., Walker E., Pogge D., Ahluvalia T., Stokes J., Handelsman L., Medrano M., Desmond D. (2003). Development and Validation of a Brief Screening Version of the Childhood Trauma Questionnaire. Child Abus. Negl..

[33] Bernstein D.P., Fink L., Handelsman L., Foote J. (1998). Childhood Trauma Questionnaire. Assessment of Family Violence: A Handbook for Researchers and Practitioners.

[34] Mackinnon A.J., Henderson A.S., Scott R., Duncan-Jones P. (1989). The Parental Bonding Instrument (PBI): An Epidemiological Study in a General Population Sample. Psychol. Med..

[35] Kroenke K., Spitzer R.L., Williams J.B.W. (2001). The PHQ-9: Validity of a Brief Depression Severity Measure. J. Gen. Intern. Med..

[36] Spitzer R.L., Kroenke K., Williams J.B., Löwe B. (2006). A Brief Measure for Assessing Generalized Anxiety Disorder: The GAD-7. Arch. Intern. Med..

[37] Blevins C.A., Weathers F.W., Davis M.T., Witte T.K., Domino J.L. (2015). The Posttraumatic Stress Disorder Checklist for *DSM-5* (PCL-5): Development and Initial Psychometric Evaluation. J. Trauma. Stress.

[38] Bernstein E.M., Putnam F.W. (1986). Development, Reliability, and Validity of a Dissociation Scale. J. Nerv. Ment. Dis..

[39] Bargai N., Ben-Shakhar G., Shalev A.Y. (2007). Posttraumatic Stress Disorder and Depression in Battered Women: The Mediating Role of Learned Helplessness. J. Fam. Viol..

[40] Condon J.T., Corkindale C.J. (1998). The Assessment of Parent-to-Infant Attachment: Development of a Self-Report Questionnaire Instrument. J. Reprod. Infant Psychol..

[41] Ayers S., Wright D.B., Thornton A. (2018). Development of a Measure of Postpartum PTSD: The City Birth Trauma Scale. Front. Psychiatry.

[42] (2023). Screening and Diagnosis of Mental Health Conditions During Pregnancy and Postpartum: ACOG Clinical Practice Guideline No. 4. Obstet. Gynecol..

[43] Costello A.B., Osborne J. (2005). Best Practices in Exploratory Factor Analysis: Four Recommendations for Getting the Most from Your Analysis. Pract. Assess. Res. Eval..

[44] Boomsma A. (2000). Reporting Analyses of Covariance Structures. Struct. Equ. Model. A Multidiscip. J..

[45] Satorra A., Bentler P.M. (2001). A Scaled Difference Chi-Square Test Statistic for Moment Structure Analysis. Psychometrika.

[46] Cohen J., Cohen P., West S.G., Aiken L.S. (2013). Applied Multiple Regression/Correlation Analysis for the Behavioral Sciences.

[47] Cohen J. (2013). Statistical Power Analysis for the Behavioral Sciences.

[48] Kraemer H.C. (1997). Coming to Terms With the Terms of Risk. Arch. Gen. Psychiatry.

[49] Jennissen S., Holl J., Mai H., Wolff S., Barnow S. (2016). Emotion Dysregulation Mediates the Relationship between Child Maltreatment and Psychopathology: A Structural Equation Model. Child Abus. Negl..

[50] Christ C., De Waal M.M., Dekker J.J.M., Van Kuijk I., Van Schaik D.J.F., Kikkert M.J., Goudriaan A.E., Beekman A.T.F., Messman-Moore T.L. (2019). Linking Childhood Emotional Abuse and Depressive Symptoms: The Role of Emotion Dysregulation and Interpersonal Problems. PLoS ONE.

[51] Hayward L.E., Vartanian L.R., Kwok C., Newby J.M. (2020). How Might Childhood Adversity Predict Adult Psychological Distress? Applying the Identity Disruption Model to Understanding Depression and Anxiety Disorders. J. Affect. Disord..

[52] Keeton C.P., Perry-Jenkins M., Sayer A.G. (2008). Sense of Control Predicts Depressive and Anxious Symptoms across the Transition to Parenthood. J. Fam. Psychol..

[53] Kestler-Peleg M. (2026). Childbirth under Prolonged and Cumulative Stress: The Roles of Perceived Risk, Self-Compassion, and Intolerance of Uncertainty in Predicting Postpartum Psychological Well-Being. Midwifery.

[54] Leite Ongilio F., Gaspardo C.M., Linhares M.B.M. (2023). Maternal History of Adversity and Subsequent Mother–Child Interactions at Early Ages: A Systematic Review. Trauma Violence Abus..

[55] O’Dea G.A., Youssef G.J., Hagg L.J., Francis L.M., Spry E.A., Rossen L., Smith I., Teague S.J., Mansour K., Booth A. (2023). Associations between Maternal Psychological Distress and Mother-Infant Bonding: A Systematic Review and Meta-Analysis. Arch. Women’s Ment. Health.

[56] Walker-Mao C., Farewell C.V., Nagle-Yang S., Blackwell S., Leiferman J.A. (2024). Adverse Childhood Experiences Predict Anxiety during Postpartum and Early Childhood Parenting. J. Psychosom. Obstet. Gynecol..

[57] Carmassi C., Conti L., Gravina D., Nardi B., Dell’Osso L. (2022). Emotional Dysregulation as Trans-Nosographic Psychopathological Dimension in Adulthood: A Systematic Review. Front. Psychiatry.

[58] Spaegele N., Lewin T., Talmon A. (2024). Trauma Symptom Patterns in a Large Sample of Military Personnel Outpatients: Differential Relations to Trauma Exposure, Depression, and Anxiety Symptoms. Psychol. Trauma Theory Res. Pract. Policy.

[59] Le Bas G., Youssef G., Macdonald J.A., Teague S., Mattick R., Honan I., McIntosh J.E., Khor S., Rossen L., Elliott E.J. (2022). The Role of Antenatal and Postnatal Maternal Bonding in Infant Development. J. Am. Acad. Child Adolesc. Psychiatry.

[60] Paucsik M., Baeyens C., Tessier D., Shankland R. (2024). Reducing Emotion Dysregulation Online in Nonclinical Population with Compassion Focused Therapy and Emotional Competencies Program: A Randomized Controlled Trial. J. Clin. Psychol..

[61] Meier J., Meine L.E., Schüler K., Wessa M. (2025). Distinct Trajectories of Perceived Control over Aversive Stimulation Predict Affective Reactions to Stressors over and above Objective Control. Sci. Rep..

[62] Brand B.L., Schielke H.J., Putnam K., Pierorazio N.A., Nester M.S., Robertson J., Myrick A.C., Loewenstein R.J., Putnam F.W., Steele K. (2025). A Randomized Controlled Trial Assists Individuals with Complex Trauma and Dissociation in Finding Solid Ground. Psychol. Trauma Theory Res. Pract. Policy.

[63] Antuña-Camblor C., Gómez-Salas F.J., Burgos-Julián F.A., González-Vázquez A., Juarros-Basterretxea J., Rodríguez-Díaz F.J. (2024). Emotional Regulation as a Transdiagnostic Process of Emotional Disorders in Therapy: A Systematic Review and Meta-Analysis. Clin. Psychol. Psychoth..

