# Supplementary material for: Beyond Trauma Exposure and Symptoms: Preconception Transdiagnostic Vulnerability and Postpartum Outcomes in Healthy Women
Source: J Clin Med. 2026 Sep 13;15(18):7104. doi: 10.3390/jcm15187104 (PMC13607174; doi:10.3390/jcm15187104)
Supplement: Supplementary file 1 [file jcm-15-07104-s001.zip › jcm-4498960-supplementary.pdf]

Supplementary Materials

Parallel Analysis for the Exploratory Factor Analysis

Parallel analysis compared observed eigenvalues against eigenvalues derived from 1,000 simulated datasets of equivalent size. The first three factors had observed eigenvalues (3.03, 1.15, and 0.31, respectively) exceeding their corresponding simulated eigenvalues, whereas the fourth factor's observed eigenvalue (0.14) fell below its simulated counterpart, supporting retention of a three-factor solution (see Figure S1 for the Scree Plot).

Supplementary Figure S1. Scree Plot of Parallel Analysis

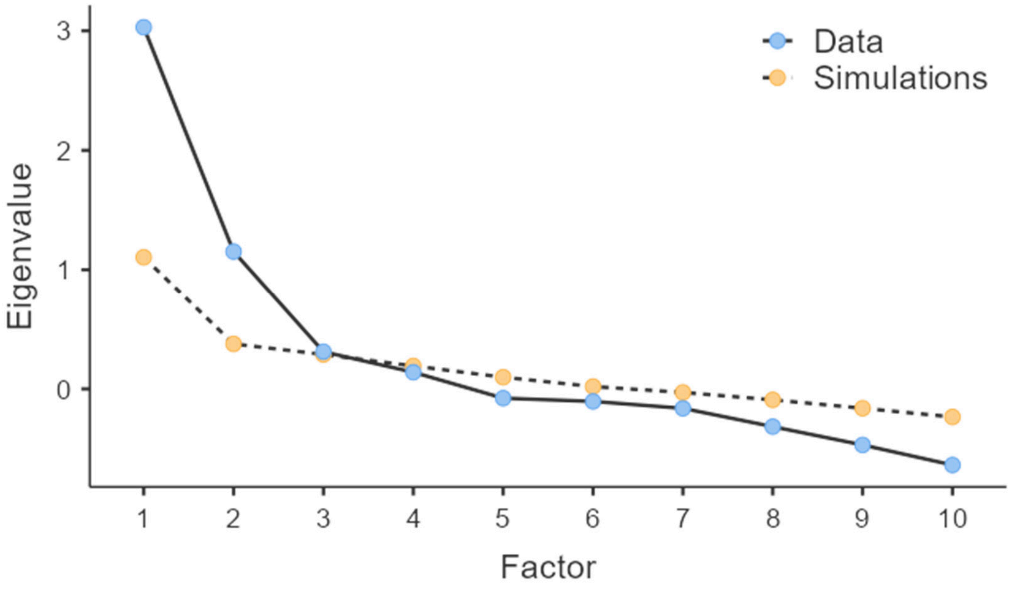

Supplementary Table S1. Structural Equation Model: Standardized Path Coefficients

Path	β	95% CI (L)	95% CI (U)	p
**Main Model: Structural Paths**				
Risk → Vulnerability	0.40	0.24	0.56	< 0.001
Vulnerability → Symptoms	0.60	0.41	0.79	< 0.001
Risk → Symptoms (direct)	-0.05	-0.21	0.11	0.57
Risk → Symptoms (indirect)	0.24	0.11	0.37	0.001
**Main Model: Measurement – Risk**				
CTQ - Neglect	0.97	0.88	1.055	-
CTQ - Abuse	0.65	0.53	0.76	< 0.001
PBI Parental Bonding (Maternal Care)	-0.70	-0.83	-0.57	< 0.001
PBI Parental Bonding (Paternal Care)	-0.60	-0.73	-0.48	< 0.001
**Main Model: Measurement – Vulnerability**				
DERS (Emotional Regulation)	0.78	0.67	0.89	-
LHQ (Learned Helplessness)	0.69	0.56	0.82	< 0.001
DES (Dissociation)	0.53	0.38	0.67	< 0.001
**Main Model: Measurement – Symptoms**				
GAD-7 Total (Anxiety)	0.85	0.75	0.95	-
PHQ-9 (Depression)	0.81	0.72	0.91	< 0.001
PCL-5 (PTSD)	0.47	0.32	0.62	< 0.001
**Exploratory Model: Learned Helplessness (LHQ)**				
Risk → LHQ	0.37	0.22	0.51	< 0.001
LHQ → Symptoms	0.44	0.30	0.59	< 0.001
Risk → Symptoms (direct)	0.03	-0.12	0.18	0.73
Risk → Symptoms (indirect)	0.16	0.07	0.25	0.002
**Exploratory Model: Emotion Regulation (DERS)**				
Risk → DERS	0.26	0.11	0.41	0.003
DERS → Symptoms	0.40	0.23	0.57	< 0.001
Risk → Symptoms (direct)	0.08	-0.08	0.23	0.33
Risk → Symptoms (indirect)	0.10	0.03	0.18	0.01
**Exploratory Model: Dissociation (DES)**				
Risk → DES	0.16	0.02	0.30	0.03
DES → Symptoms	0.28	0.11	0.46	0.002
Risk → Symptoms (direct)	0.13	-0.01	0.28	0.07
Risk → Symptoms (indirect)	0.04	0.00	0.09	0.06

Table S1. Exploratory LHQ, DERS and DES models N = 160; Reference indicators fixed at 1.0 for identification (denoted by -). Indirect effects represent the product of path coefficients through the mediator. CTQ, Childhood Trauma Questionnaire; PBI, Parental Bonding Instrument; GAD-7, Generalized Anxiety Disorder; PHQ-9, Patient Health Questionnaire; PCL-5, PTSD Checklist for DSM-5; DERS, Difficulties in Emotion Regulation Scale; DES, Dissociative Experiences Scale; LHQ, Learned Helplessness Questionnaire.

Supplementary Table S2. Full Hierarchical Regression Statistics Predicting Postpartum Outcomes

MPAS (N=130)															
**Step**	**Predictor**	**B**	**SE**	**t**	**p**	**β**	**Model R^2**	**Adj. R^2**	**F**	**df1**	**df2**	**p (model)**	**ΔR^2**	**F-change**	**p (change)**
1	Risk	−3.36	0.74	−4.51	<0.001	−0.37	0.14	0.13	20.4	1	128	<0.001			
2	Risk	−2.53	0.77	−3.31	0.001	−0.28	0.20	0.19	15.8	2	127	<0.001	0.06	9.90	0.002
2	Vulnerability	−2.37	0.75	−3.15	0.002	−0.27									
3	Risk	−2.43	0.77	−3.17	0.002	−0.27	0.21	0.19	11.3	3	126	<0.001	0.01	1.95	0.16
3	Vulnerability	−1.84	0.84	−2.21	0.03	−0.20									
3	Symptoms	−1.20	0.86	−1.40	0.16	−0.13									

BiTS (N=130)															
**Step**	**Predictor**	**B**	**SE**	**t**	**p**	**β**	**Model R^2**	**Adj. R^2**	**F**	**df1**	**df2**	**p (model)**	**ΔR^2**	**F-change**	**p (change)**
1	Risk	1.89	0.76	2.47	0.01	0.21	0.04	0.04	6.08	1	128	0.01			
2	Vulnerability	2.90	0.76	3.83	<0.001	0.33	0.14	0.13	10.69	2	127	<0.001	0.10	14.6	<0.001
3	Risk	0.46	0.70	0.66	0.51	0.05	0.32	0.30	19.38	3	126	<0.001	0.17	31.6	<0.001
3	Vulnerability	1.00	0.76	1.31	0.19	0.11									
3	Symptoms	4.40	0.78	5.62	<0.001	0.48									

PHQ-9 (N=133)															
**Step**	**Predictor**	**B**	**SE**	**t**	**P**	**β**	**Model R^2**	**Adj. R^2**	**F**	**df1**	**df2**	**p (model)**	**ΔR^2**	**F-change**	**p (change)**
1	Risk	0.80	0.36	2.19	0.03	0.19	0.03	0.03	4.78	1	131	0.03			
2	Vulnerability	1.19	0.36	3.27	0.001	0.29	0.11	0.09	7.92	2	130	<0.001	0.07	10.7	0.001
3	Risk	0.29	0.36	0.81	0.42	0.07	0.20	0.18	10.93	3	129	<0.001	0.09	15.2	<0.001
3	Vulnerability	0.48	0.39	1.24	0.22	0.12									
3	Symptoms	1.53	0.39	3.90	<0.001	0.36									

GAD-7 (N=134)															
**Step**	**Predictor**	**B**	**SE**	**t**	**p**	**β**	**Model R^2**	**Adj. R^2**	**F**	**df1**	**df2**	**p (model)**	**ΔR^2**	**F-change**	**p (change)**
1	Risk	0.79	0.33	2.41	0.02	0.20	0.04	0.03	5.82	1	132	0.017			
2	Vulnerability	1.46	0.32	4.62	<0.001	0.39	0.18	0.16	14.03	2	131	<0.001	0.13	21.3	<0.001
3	Risk	0.17	0.30	0.58	0.56	0.04	0.30	0.28	18.69	3	130	<0.001	0.12	23.3	<0.001
3	Vulnerability	0.72	0.33	2.19	0.03	0.19									
3	Symptoms	1.60	0.33	4.82	<0.001	0.41									

Table S2. Hierarchical linear regression models examining associations between latent Risk, Vulnerability, and Symptoms factors and postpartum outcomes (maternal bonding, traumatic birth symptoms, postpartum depression, and postpartum anxiety). Predictors were entered sequentially: Step 1 = Risk factor; Step 2 = Vulnerability factor; Step 3 = Symptom factor. B, unstandardized coefficient; SE, standard error; β, standardized coefficient; Adj. R^2, adjusted R^2; ΔR^2, change in R^2 associated with the entry of each step; F-change, F statistic for the change in R^2. Blank cells for Steps 1–2 in the BiTS, PHQ-9, and GAD-7 models reflect that only final-step (Step 3) coefficients are reported at the predictor level; model fit statistics are reported for all steps. MPAS, Maternal Postnatal Attachment Scale; BiTS, Birth Trauma Scale; PHQ-9, Patient Health Questionnaire; GAD-7, Generalized Anxiety Disorder.
